# Assessment of Platelet Indices in Patients with Neurodegenerative Diseases: Mean Platelet Volume Was Increased in Patients with Parkinson's Disease

**DOI:** 10.1155/2013/986254

**Published:** 2013-12-08

**Authors:** Abdulkadir Koçer, Aslı Yaman, Elvin Niftaliyev, Hümeyra Dürüyen, Mehmet Eryılmaz, Emel Koçer

**Affiliations:** ^1^Medical Faculty, Istanbul Medeniyet University, Istanbul, Turkey; ^2^Düzce University, Düzce, Turkey; ^3^Bezmialem Vakıf University, Istanbul, Turkey

## Abstract

Platelets induce chronic inflammation which is a key step in atherosclerosis and may be involved in the progression of neurodegenerative diseases (NDD). We aimed to measure the mean platelet volume (MPV) and platelet count (PLC) in NDD patients. The present study was designed to investigate the platelet function by measuring MPV and PLC in NDD. A total of 182 outpatients with Alzheimer's (AD) or Parkinson's diseases (PD) were included. The control group consisted of 104 healthy subjects. Platelet count was similar between groups. MPV values of PD patients were higher than those of AD patients and controls (*P* < 0.001). MPV correlated negatively with Heohn and Yahr scale (HYS) score (*P* < 0.001). Increased MPV in patients with PD may point to a platelet dysfunction. High-grade inflammation presents with low levels of MPV as seen in PD patients with high HYS scores.

## 1. Introduction

Alzheimer's disease (AD) and Parkinson's disease (PD) are the most common progressive neurodegenerative disorders (NDD) in the elderly, and vascular risk factors may be involved in the pathogenesis of both diseases [1, 2]. It is well known that the disrupted microvascular integrity in brain parenchyma may play a role in the chain of events ending with AD [1–6]. Besides atherosclerosis, many reports suggest that inflammation may be involved in neurodegenerative disorders [7]. Amyloid beta 42 plays a central role in the neurodegenerative process and triggers inflammation in AD. Although molecular basis is not known, interleukins and cytokines may induce the activation of signaling pathways leading to further inflammation, neuronal injury, and later neuronal death [7]. Nonsteroidal anti-inflammatory drug use may decrease the incidence of AD and this knowledge supports the role of inflammation in AD pathogenesis [7]. On the other hand, there are conflicting results about PD. The commonly known risk factors are less frequently seen in the patients with PD [8]. Cumulative incidence of ischemic stroke and myocardial infarction is less frequent in PD patients too [9, 10]. In contrast, some new articles reported that cardiovascular risk factors, such as diabetes mellitus and central obesity, have been associated with Parkinson's disease (PD), but data on blood pressure and PD are still lacking [2, 11, 12]. Qiu et al. suggested that hypertension was associated with an increased risk of PD and optimal control of blood pressure might reduce the incidence of PD in women [2, 11, 12]. Also we know that the treatment with levodopa increases blood homocysteine, and high levels of homocysteine may be associated with the increased incidence of vascular events in PD [13].

Platelet-induced chronic inflammation is a key step in atherosclerosis and promotes plaque development, intimae hyperplasia, and restenosis [14, 15]. A range of molecules, present on the platelet surface and/or stored in platelet granules, contributes to the cross-talk of platelets with other inflammatory cells during the vascular inflammation involved in the development and progression of atherosclerosis [16, 17]. During the prolonged course of atherosclerotic disease, platelets have an important role not only in the initiation but also in the progression and exacerbations of atherosclerotic disease [15]. Platelet activity can be conveniently estimated by measuring the mean platelet volume (MPV) and platelet count (PLC) [18].

We hypothesized that platelets might have an important role in the development of NDD if there were relationships between atherosclerosis, inflammation, and platelets. To address this, we selected the most commonly known two-different NDD groups, that is, AD as a prototype of cortical involvement and PD as a prototype of subcortical involvement. We used a case-control method to compare the serum MPV and PLC in NDD patients with those in controls.

## 2. Methods

Patients (*n* = 385) diagnosed with AD and PD were enrolled from January 2010 until September 2012 in the Departments of Neurology at Düzce University Medical Faculty and Bezmialem Vakif University Medical Faculty Hospitals. Data were obtained by clinical interview, physical and neurological examinations in outpatient clinics specialized in neurodegenerative disorders, laboratory exams, and computed tomography or magnetic resonance imaging. Three hundred thirty-five patients who accepted to be involved in the study and met the diagnostic criteria of probable AD and PD according to the established criteria were evaluated [19–21]. Participants who had cancer, abnormal liver and thyroid function tests moderate and severe level of hypertension (≥160/100 mmHg), uncontrolled diabetes (HbA1C > 8), high levels of total cholesterol (>200 mg/dL) with BMI > 30, any other dementia including vitamin B12 deficiency, and previous history of stroke and receiving antiplatelet or anticoagulant therapy and those who were active smokers (smoking 1 pack or more per day) and heavy drinkers were excluded. After the evaluation with the exclusion criteria, 182 outpatients with AD (*n* = 89) and PD (*n* = 93) were included in the study. The Turkish version of the minimental state examination (MMSE) was used to screen for cognitive impairment of AD patients [22, 23]. PD patients were examined by using Hoehn and Yahr scale (HYS) which is a scale for PD's severity that rates between 0 and 5 based on the level of clinical disabilities [24]. The control group consisted of 104 subjects, who were matched for age and sex without dementia, PD, any other neurodegenerative diseases, stroke, and hematologic disease [25]. Mean blood pressure levels were recorded after a 7-day followup and repeated measurements. Then, the modifiable risk factors determined by physical examination or self-reported current use of antihypertensive medication, cholesterol lowering drugs, or antidiabetic agents were written down in all participants. In addition, drugs used for AD and PD or other drugs such as antidepressants, which were widely used in this population, were recorded as well. The study protocol was approved by the local ethics committee. The aims and procedures of the investigation were orally explained and a consent form was taken from all participants or relatives.

After the 12-hour fasting, ten milliliters of blood samples was drawn from the antecubital vein in the early morning (between 9:00 and 10:00 a.m.), and the samples were analyzed at 13:00 p.m. We measured the MPV and platelet count in a blood sample collected in citrate (1 : 4 vol/vol) to avoid the platelet swelling induced by EDTA. The expected values for PLC and MPV in our laboratory ranged from 140 to 450 × 10^3^/*μ*L and from 7 to 11 fL, respectively. Laboratory exams included complete blood count, chemical profile, blood fasting glucose, total cholesterol, thyroid stimulating hormone, and vitamin B12 levels.

## 3. Statistical Analyses

Descriptive analyses were presented by using means and standard deviations for normally distributed variables. ANOVA test was used to compare normally distributed variables (MPV) between the patients and control groups. Descriptive analyses were presented by using medians and Kruskal Wallis test was used to compare nonnormally distributed variables (PLC and all other variables shown in Table 1) between the patients and control groups. Spearman's correlation analysis was made to evaluate the relationships between MPV and the other findings in PD and AD patients. Data were analyzed to identify whether MPV was independently associated with age, blood glucose, cholesterol and thyroid stimulating hormone levels, PLC, and HYS score by using univariate logistic and stepwise multivariate logistic regression models in PD. Odds ratios and 95% confidence intervals were estimated for the effect of independent variables. A *P* value <0.05 was accepted to be statistically significant.

## 4. Results

**Figure 1:**
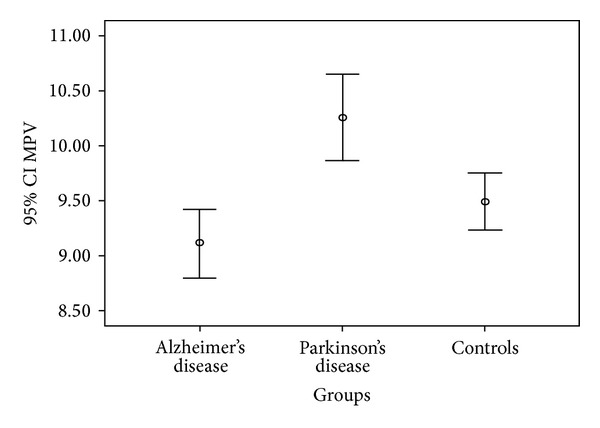
Comparison of mean platelet volume (MPV) values.

One hundred and forty-one participants were female and 145 were male. Demographic and clinical characteristics of patients and controls participating in the study are shown in Table 1. Mean PLC was similar between groups. However, MPV values of PD patients were higher than those of AD patients and controls (Figure 1, *P* < 0.001). Mean MPV values of PD, AD, and controls were 10.26 ± 1.91, 9.11 ± 1.48, and 9.49 ± 1.33 f/L, respectively (*F*: 20.77, *P* < 0.001). The mean differences between PD and AD and controls were calculated as 1.48 and 1.09 fL, respectively (*P* < 0.001). The presence of risk factors was similar between PD and AD groups (Table 1).

In AD patients, MMSE minimental state examination score was 17.56 ± 4.68 and the analysis of the relationships between MMSE scores and MPV or platelet counts did not reveal any significant result. By Spearman analysis, MPV correlated with the PLC (*r*: –0.38, *P* < 0.001), age (*r*: −0.21, *P* = 0.04), presence of hypercholesterolemia (*r*: −0.22, *P* = 0.038), and presence of antidepressant use (*r*: 0.25, *P* = 0.017) in AD patients. The other relationships between MPV or PLC and presence of hypertension or hyperglycemia, the other drugs used for AD and gender were nonsignificant.

In the statistical analysis of PD patients, MPV correlated with the PLC (*r*: –0.36, *P* < 0.001), HYS score (*r*: −0.32, *P* = 0.002), and presence of antidepressant use (*r*: 0.31, *P* = 0.003). The relationship between MPV and age was not significant (*P* > 0.05). There was a positive correlation between MPV value and the presence of hypercholesterolemia (*r*: −0.26, *P* = 0.01). The other relationships between MPV or PLC and other vascular risk factors, gender and treatment regiments related to PD were not significant. In stepwise multivariate analysis, PLC (Beta value = −0.38, *P* = 0.001) and HYS score (Beta value = −0.39, *P* = 0.002) independently predicted MPV in the patients with PD.

## 5. Discussion

Peripheral blood cells, such as platelets or lymphocytes, have been widely studied in several neuropsychiatric disorders previously [26]. Platelets and their secreted products intervene in inflammation and tissue regeneration [14]. In addition, platelets are the main source of 5-hydroxytryptamine (5-HT) and contain all the enzymatic machinery to amyloid precursor protein (APP) processing [27]. Platelet dysfunction and APP processing abnormalities are believed to occur rather early during the course of AD [1, 28, 29]. Similarly, Inestrosa et al. reported that platelets were elevated in comparison to nondemented healthy individuals and concluded that platelets might provide a systemic marker of AD [30]. In vascular dementia, PLC showing platelet activity was decreased and MPV was increased [31]. However, there are conflicting results about MPV in AD patients. Wang et al. reported decreased MPV level, but Yesil et al. reported increased MPV level in AD [32, 33]. It is well known that high MPV associates with low-grade inflammatory conditions [34]. Moreover, increased MPV has been reported in patients with myocardial infarction and vascular risk factors [35, 36]. High-grade inflammatory diseases, such as active rheumatoid arthritis or attacks of familial Mediterranean fever, present with low levels of MPV [34]. Our study focused on platelets, MPV, and PLC in AD patients. MPV of AD patients was lower than that of controls, but the difference was not significant. High-risk patients with suspected atherosclerosis were strictly excluded in the present study. Therefore, we think that decreased MPV supported the role of high-grade inflammation in AD patients. Further studies are needed to explore these conflicting results reported in the literature.

Inflammation is also a neuropathological feature of the Parkinsonian brain. It is believed that the activated glial cells, which compose the majority of this inflammatory response, contribute to the neurodegenerative process through the production of toxic molecules [37]. Longstanding debate with toxicity and oxidative stress is suggested as a cause in PD [38, 39]. In the present study, we found that the MPV of PD patients was significantly higher than those of controls and AD patients (Table 1). Platelets play a crucial role in atherosclerosis and stroke [16–18, 34–36]. The size of the platelets, represented by MPV, has been shown to be closely related to their reactivity, and large platelets are metabolically and enzymatically more active than the small ones, having a higher thrombotic potential [34–36, 40]. Confounding effects on MPV such as patients carrying high risk related to atherosclerosis were carefully excluded in the present study [41]. Because of these strict exclusion criteria, we believe that our finding supports the fact that MPV may be a risk factor for atherosclerosis in the patients with PD. We also found a negative correlation between MPV and PLC as reported previously [34–36, 40]. We have found that antidepressant use was inversely correlated with MPV. This finding was similar to several previous reports suggesting an antiplatelet effect of antidepressant medications [42–44]. In critics, it was difficult to exclude the patients with antidepressant use because a lot of patients were using antidepressants and had depression in both groups. Another important finding of our study was that the progressed PD was correlated with MPV negatively. Decreased MPV during the progression supported the inflammation theory in PD and these findings suggested that there was another problem other than inflammation in the early periods of idiopathic Parkinsonism. To the best of our knowledge, these findings have not been reported previously. So we believe that our results are valuable in clinical followup and decreased MPV reflects inflammation in the pathological stages of PD.

## Figures and Tables

**Table 1:** Demographic and clinical characteristics of participants.

Characteristics	Group	Median	Min–Max	*P* value
Gender (male/female)	AD	40/49*		0.25
	PD	46/47*		
	Control	59/45*		

Age (year)	AD	75	46–88	0.052
	PD	73	55–87	
	Control	72	60–86	

Platelet count × 10^3^/mm^3^	AD	237	122–467	0.64
	PD	230	102–561	
	Control	250	143–470	

Fasting blood glucose level (mg/dL)	AD	103.5	80–184	0.56
	PD	104	77–271	
	Controls	110.5	75–288	

Total cholesterol level (mg/dL)	AD	114	86–174	0.06
	PD	105	66–172	
	Controls	95	79–172	

Number of the patients with hypertension (+/−)	AD	29/60*		0.003
	PD	38/55*		
	Control	15/89*		

Number of the patients with hyperglycemia (+/−)	AD	18/71*		0.004
	PD	25/68*		
	Control	6/98*		

Number of the patients with hypercholesterolemia (+/−)	AD	7/82*		0.81
	PD	16/77*		
	Control	10/94*		

Number of the patients with antidepressant use (+/−)	AD	35/54*		<0.001
	PD	34/59*		
	Control	0/98*		

MMSE score	AD	18	6–26
HYS score	PD	2	1–4

*Number of the patients; (+/−): present/not present; MMSE: minimental state examination; HYS: Hoehn and Yahr scale; AD: Alzheimer's disease; PD: Parkinson's disease.
